# Associated Factors of Exclusive Breastfeeding Intention among Pregnant Women in Najran, Saudi Arabia

**DOI:** 10.3390/nu15133051

**Published:** 2023-07-06

**Authors:** Heba A. Ibrahim, Mohammed A. Alshahrani, DaifAllah D. Al-Thubaity, Samiha H. Sayed, Sultan A. Almedhesh, Wafaa T. Elgzar

**Affiliations:** 1 Department of Maternity and Childhood Nursing, Nursing College, Najran University, Najran 66271, Saudi Arabia; heaibrahim@nu.edu.sa (H.A.I.);; 2 Department of Clinical Laboratory Sciences, College of Applied Medical Sciences, Najran University, Najran 66271, Saudi Arabia; 3 Department of Public Health, College of Health Sciences, Saudi Electronic University, Damman 34223, Saudi Arabia; 4 Department of Community Health Nursing, Faculty of Nursing, Damanhour University, Damanhour 22511, Egypt; 5 Department of Pediatrics, College of Medicine, Najran University, Najran 66271, Saudi Arabia

**Keywords:** attitude, breastfeeding, intention, knowledge, Saudi Arabia

## Abstract

The exclusive breastfeeding (EBF) intention conceived by pregnant women is the most important predictor of breastfeeding (BF) initiation, duration, and continuation. This study explores the associated factors of EBF intention among pregnant women. This was a descriptive cross-sectional study conducted from November 2022 to January 2023 with 382 pregnant women who came to the outpatient clinic in the Maternal and Children Hospital (MCH). Four instruments were used for data collection: the Infant Feeding Intention scale, the Gender-Friendly BF Knowledge scale (GFBKS), the Iowa Infant Feeding Attitude scale (IIFAS), and the basic data questionnaire. The study findings indicated that 51.8% and 75.9% of gravida women had adequate knowledge and a positive attitude regarding BF. Furthermore, 56.3% of the participants had a high intention for EBF. Binary logistic regression illustrated that occupational status, antenatal care, plan for the current pregnancy, BF practice, last child delivery mode, medical disorder during the current pregnancy, age, BF knowledge, and attitude are potential predictors. The goodness of fit test revealed that 46.8% of the EBF intention could be anticipated through the positive pre-mentioned factors. The low EBF intention is modifiable by addressing the previously positive predictors. BF educational interventions should be tailored based on EBF intention predictors in order to be effective and lead to behavior change.

## 1. Introduction

The World Health Organization (WHO) defined Exclusive Breastfeeding (EBF) as giving only breast milk to an infant until six months of life [1]. During EBF, oral rehydration therapy, vitamins, minerals, and other prescribed medications are allowed, but water and herbal teas are not allowed as breast milk is the sole infant nutrient. Breast milk can be given directly from the breast or through expressed milk for hospitalized infants [2]. 

Breast milk has a unique composition that makes it ideal to meet all infant needs up to six months, half of its need until one year, and one-third of the nutritional requirement in the second year [1]. Surprisingly, BM composition is not the same for the whole two years [3,4]. The BM proteins, carbohydrates, lipids, and other component quantity and quality varied considerably over a single feed and over two years to compensate for the infants’ requirements. In addition, BM contains biologically active compositions that can guide the infant’s immunological system and intestinal microbiota. Furthermore, breast milk differs from one woman to another based on her genotype [5]. The human milk component is affected by many factors, including age, BF duration, and women’s general health. Breastmilk, especially colostrum, contains high levels of antioxidants, growth factors, and cytokines. The breastmilk active component was found at a higher level in the case of preterm infants compared with term infants [6]. 

EBF has unquestionable benefits for infants and mothers. It can reduce the risk of gastrointestinal infection, diarrheal disease, and other infectious diseases by sharing antibiotics from the mother to the infant [7]; BF provides complete nutritional requirements, improved infant IQ, and decreased risk for infant obesity and chronic illness such as diabetes, hypertension, cardiovascular diseases, hyperlipidemia, and cancer in later life [8]. BF women have more rapid maternal weight loss postpartum [9], which delays the return of menstruation and allows for delayed fertility [10]. Caik-Ksepka et al. performed a review to identify the association between locational amenorrhea, fertility, and bone metabolism. They concluded that although bone loss during lactation is a physiological event that meets the infant’s needs for calcium, bone rebuilding occurs after weaning, and BF is not associated with osteoporosis during post-menopause [10]. BF also decreases the risk for long-term chronic illnesses such as diabetes mellitus, cardiac diseases, obesity, hyperlipidemia, and some type of cancer [8]. Moreover, the perinatal emotional condition has a significant effect on the breastfeeding practice. Stuebe et al. performed longitudinal research to explore the relationship between the prenatal mood and psychological state of pregnant women and BF. They found that depression and anxiety symptoms during pregnancy were associated with negative BF outcomes and early cessation of BF. They also found that pregnant women with a history of childhood trauma, eating disorder symptoms, and poor sleep quality before childbirth were at increased risk. Therefore, they recommended targeted support in these areas to promote EBF [11].

The prevalence of EBF varies by country and region. WHO stated that although EBF is strongly recommended, only less than half of infants worldwide have to breastfeed exclusively [1]. In Saudi Arabia, a systematic review by Al Junaid et al. showed inconsistent data associated with the prevalence and predictors of EBF. Its prevalence varies between and within the various regions of Saudi Arabia, ranging from 0.8% to 43.9% among 17 studies involved in the review [12]. In addition, Alzaheb et al. conducted a study to investigate the associated factors with EBF in Tabuk, Saudi Arabia, and found that EBF was practiced by 31.4% of their study participants [13].

The EBF intention conceived by a pregnant woman is the most important predictor of BF initiation, duration, and continuation. The greater the intention to breastfeed during pregnancy, the more likely a mother is to breastfeed exclusively after childbirth [14]. Although BF is a physiological process concerning the mother and her neonate, societal and cultural legacies also contribute to its success. Hence, assessing the associated factors of the EBF intention should be viewed from numerous perspectives [15].

A Saudi study conducted by Alnasser et al. at King Saud University Medical Center reported that pregnant Saudi women have insufficient knowledge regarding the importance of EBF. In addition, their intentions to EBF were associated with their knowledge and attitude toward BF [16]. Therefore, it is necessary to explore whether maternal characteristics may influence BF intention during the prenatal period. When we can predict the pregnant women’s intention for EBF, proper support can be delivered to enhance BF initiation and continuation. Furthermore, data provided from the current study can provide a strong base to build effective BF educational interventions. Therefore, the current study explores the associated factors of EBF intention among pregnant women. 

## 2. Methods

### 2.1. Study Design

This cross-sectional study explores the predictors of EBF intention among pregnant women.

#### 2.1.1. Operational Definition for Terms

EBF was defined as giving the baby only BF for up to six months of life [17]. Nonexclusive BF was defined as the introduction of any supplementary feeding, including water and herbs, during the first six months of the infant’s life [17].

#### 2.1.2. Setting and Participants

The study was executed in an antenatal follow-up clinic in the Maternal and Children Hospital (MCH) in Najran, Saudi Arabia. MCH is a specialized hospital in the Najran area. Thus, it provides obstetrics care for a large proportion of the population. Najran is a large city in southwestern Saudi Arabia on the border with Yemen. Saudi Arabia is considered a high-income country, and its residents have various customs and beliefs concerning maternal and child health in general and BF more specifically. These traditions and beliefs may have a significant effect on EBF intention [18].

Inclusion criteria were pregnant Saudi women free from contraindications for BF such as HIV, hepatitis B, and breast surgery, aged 18 years and older, free from hearing disabilities, and accepting participation. 

### 2.2. Sample Size Determinations and Sampling Procedures

**Figure 1:**
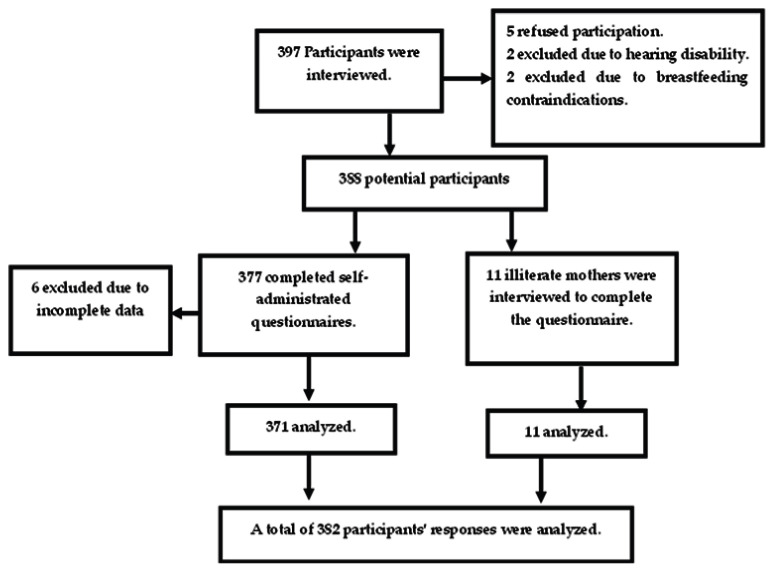
Participants flow chart.

The sample size was estimated utilizing the single population proportion formula:

(1) $$\frac{n={\left(Z1-\alpha /2\right)}^{2}P(1-P)}{d^{2}}$$

where n is the sample size; Z1 − α/2 is the standard normal variable, at 95% confidence degree = 1.96; P is the anticipated proportion of pregnant women intended to breastfeed (P = 65.4%) from previous research [19]; and d is the margin of error = 0.05. The result of the sample size calculation equals 382 after the addition of 15% to anticipate the low response rate. A systemic random sampling procedure was utilized to recruit pregnant women during antenatal visits. The following chart illustrates the participants’ distribution (Figure 1).

### 2.3. Sampling Procedures

In the current study, a researcher utilized systemic random sampling. The four antenatal clinics’ daily average follow-up rate was 80 cases in both morning and afternoon shifts, according to the clinic nurses. Two researchers and two trained data collectors comprised the data collection team. Each data collector demonstrated the ability to collect 5 cases per day. A total of 20 cases can be collected by the research team daily; therefore, the sampling interval is 80/20 = 4. A random starting point is selected daily from 1, 2, 3, or 4 using a random ball then a sampling interval of 4 is applied. The data collection team was present three days per week in the waiting area for two months, from November 2022 to January 2023, until the sample size was reached. If the selected mother was illiterate, an interview was conducted, and if she was ineligible for the study, a subsequent woman was involved, then the sampling interval was kept. 

### 2.4. Measurement Tools

The study’s dependent variable is the pregnant women’s intention to breastfeed. The Infant Feeding Intention (IFI) scale was created to evaluate the maternal EBF intention during pregnancy. It was created by Nommsen-Rivers and Dewey in 2009. The IFI scale comprises five statements rated on a 4-point Likert scale ranging from 0 (very much disagree) to 4 (very much agree) except for the first statement, which is reversed score. The overall IFI score is computed by obtaining the average score of the first two statements and summing it with the remaining three statement scores; thus, the overall scale score ranges from 0–16 [20]. The IFI scale revealed has high internal consistency (r = 0.90) [21]. Also, the internal consistency reliability of the IFI Arabic version was (0.82) when tested on pregnant Lebanese women [22]. A low BF intention was considered at a score of 0–8, and a high intention was considered at 9–16. 

The study’s independent variables are BF knowledge, BF attitude, demographic characteristics, and obstetric and BF history. 

Mother BF knowledge was assessed using the GFBKS developed by Gupta et al. The scale consists of 18 statements based on the conceptual framework. It is rated on a 5-point Likert scale to evaluate the woman’s BF knowledge level. The statement scale scored as false (1), may be false (3), do not know (3), may be true (4), and true (5). The content validity for each of the 18 statements in the instrument was more than 0.80 [23]. Tamim et al. (2016) validated the Arabic version of GFBKS and reported satisfactory internal consistency (r = 0.652) [24]. The overall GFBKS score ranged from 18 to 90. Inadequate knowledge was considered when the mother’s score ranged from 18–54, and adequate knowledge when the score ranged from 55–90. 

Breastfeeding attitude was evaluated using IIFAS, developed by De la Mora et al. IIFAS comprises 17 statements rated on a 5-point Likert scale, from 1 (strongly disagree) to 5 (strongly agree). The overall IIFAS score ranges from 17 to 85, with the highest score indicating a more positive attitude. The internal consistency for all items on the scale ranged from 0.85 and 0.86 [25]. In addition, the Arabic IIFAS version was validated by Charafeddine et al. and showed satisfactory internal consistency (r = 0.640) [26]. Another Saudi study evaluated the Arabic version of IIFAS and showed accepted internal consistency (r = 0.595) [27]. The negative attitude was calculated at the IIFAS score (17–51) and the positive attitude (52–85).

Women’s demographic characteristics and obstetric history were assessed using a basic data questionnaire. It was developed by the researchers and included two parts. Part one includes demographic data such as age, residence, occupational status, educational level, husband’s educational level, and monthly income. Part two includes obstetric and BF history as gestational age for current pregnancy, antenatal care (ANC) visits, gravidity, current pregnancy planning, previous BF practice, mode of delivery of the last child, number of living children, parity, and pregnancy-related disorders. 

### 2.5. Data Collection Procedures

The data collection was carried out from November 2022 to January 2023. The data collectors clarified the study’s aim to the potential participants and took their informed consent. If the participants could read and write, a self-administered questionnaire was provided, and the data collector was present for clarification. An interview was conducted in a quiet room for the eleven illiterate participants, and the data collector filled out the interview schedule. After the interview or questionnaire completion, the basic data was confirmed from the medical record. 

### 2.6. Data Quality Control

Two of the data collectors were associate professors of maternity and childhood nursing with a previous history of data collection and research. The other two were bachelor’s degrees in nursing with prior data collection experience as they participated in numerous previous data collection procedures. Two training sessions were conducted for the data collectors to explain the questionnaire, clear up any misunderstandings, and explain the research. Illiterate participants were interviewed by the researchers only. During the data analysis, the questionnaires were IIFAS properly evaluated, and the entire questionnaire was excluded if any missing data was noticed.

### 2.7. Ethical Approval

Before commencing data collection, the research proposal and data collection instruments were assessed by the ethical committee of Najran Health Affairs (IRB Log Number 2023-02E) following approval from the deanship of scientific research. The study was also authorized by the MCH administration. Informed consent was obtained from all participants prior to data collection, and their anonymity was maintained. The study was approved by the ethical committee.

### 2.8. Data Analysis

IBM version 22 was used to analyze data. Descriptive statistics as numbers, percentages, and mean ± SD were performed to represent descriptive data. The total BF knowledge, attitude, and intention were obtained by summing items. Binary logistic regression was conducted to examine the predictors of EBF intention. Concerning the independent variables, age, total BF knowledge, total BF attitude, gravidity, and number of living children are numerical. The other variables were categorical such as residence, occupational status, level of education, husband’s educational level, monthly income, gestational age, ANC, gravidity, plan for the current pregnancy, BF practice, mode of delivery of the last child, and medical disorder during the current pregnancy. For all categorical variables, the first category was taken as a reference, and before regression analysis, the multicollinearity was checked. The model goodness of fit test was checked by the Cox and Snell R Square test. Statistically significant results were considered at *p* ˂ 0.005.

## 3. Results

### 3.1. Participants’ Basic Data

A total of 382 questionnaires out of 397 were analyzed, with a response rate of 96.2%. The mean age of the participant was 27.74 ± 6.74 years, and more than three-quarters (77.0%) of them ranged from 20–35 years. In terms of residence, the majority (94.8%) were residents in urban areas. In addition, more than half (55.5%) of them were workers, and around half (49.5%) had received secondary education. Around one-half (56.3%) of the pregnant women reported enough income (Table 1).

### 3.2. Participants’ Obstetric and BF History

Among all 382 participants, 42.1% were in the third trimester of pregnancy, and most (85.3%) of them received regular ANC. Around two-thirds of the participants were multigravida, and 60.7% planned for the current pregnancy. Only 13.4% had pregnancy-related complications. Of all 251 multigravida participants, 55.4% delivered vaginally, and 26.7% had a history of EBF. The mean number of living children was 2.40 ± 2.12 children (Table 2).

### 3.3. Participants’ BF Knowledge, Attitude, and Intention

The total evaluation of participants’ knowledge, attitude, and intention toward BF is illustrated in Table 3. The findings showed that around half (51.8%) of participants had adequate BF knowledge. At the same time, 75.9% and 56.3% of them had a positive attitude and high intention for EBF, respectively.

### 3.4. The Predictors of EBF Intention

Binary logistic regression illustrated that our model could predict 46.8% of EBF intention, where occupational status, ANC, plan for current pregnancy, BF practice, mode of delivery of the last child, pregnancy-related complications, BF knowledge, and attitude were potential predictors. Housewives had a four times higher probability of having high EBF intention than working mothers [AOR = 4.216 (1.906–9.326), *p* = 0.000]. In addition, regular ANC increased the probability of having higher EBF intention when taking irregular ANC as a reference [AOR = 1.718 (0.631–4.678), *p* = 0.028]. Planned pregnancy increased the probability of high EBF intention when taking unplanned pregnancy as a reference [AOR = 1.885 (0.966–3.676), *p* = 0.042]. Mothers who had previous experience with EBF or nonexclusive BF had a higher probability for EBF intention when compared with mothers who never breastfed [AOR = 4.284 (1.896–9.679), *p* = 0.000] and [AOR = 2.284 (1.652–7.234), *p* = 0.005], respectively. Furthermore, women with a history of vaginal delivery had a higher EBF intention when taking nulliparous women as reference [AOR = 6.628 (1.862–23.586), *p* = 0.003]. Also, being free from pregnancy-related complications is a positive predictor for higher EBF intention [AOR = 3.923 (1.297–11.867), *p* = 0.015]. Finally, having adequate knowledge and a positive attitude toward BF were positive predictors for higher EBF intention [AOR = 1.193 (1.183–1.421), *p* = 0.009] and [AOR = 1.282 (1.205–1.365), *p* = 0.000], respectively (Table 4).

## 4. Discussion

The Saudi Ministry of Health has made a great effort to enhance population awareness regarding different public health issues, including BF. BF is the key and sole nutrient for an infant under six months of age [12]. Furthermore, women’s knowledge and attitude regarding BF play a key role in EBF intention. Women’s BF knowledge, attitude, and intention should be evaluated and managed early in pregnancy to guarantee early initiation and continuation of EBF practice. In the present study, more than one-half of the participants had adequate knowledge regarding BF. Elgzar et al. reported a slightly higher rate (68.2%) of adequate knowledge in Najran, Saudi Arabia, than in the current study [28]. The difference between the two studies may be due to the study sample, where Elgzar et al. studied lactating women, while the current study investigates pregnant women; among them, 34.3% are primiparous women. It is expected that primiparous women may have lower knowledge regarding BF when compared with lactating mothers who already have BF experience. Furthermore, Hegazi et al. reported that most of their study participants had good knowledge regarding BF except for colostrum and continuation [29]. Other Saudi studies also reported good BF knowledge [16,30]. Although the BF knowledge in the current study was to some extent lower than in the previously mentioned Saudi studies, it was still much higher than in some international studies [31,32]. On the international level, Dukuzumuremyi et al. 2020 conducted a systemic review, including studies published between 2000 and 2019, and discussed BF knowledge and attitude. They found that although 84.4% of their participant were aware of EBF, the practice rate was still much lower than recommended by WHO and recommended intensive breastfeeding educational programs during the antenatal and postnatal period [33].

Attitude toward BF may be the key motivator to seek knowledge and have high BF intention. In the current study, about three-quarters of the participant had a positive attitude toward BF. Saudi Arabia is a Muslim country where the legislature is taken from the Holy Quran and Hadiths. The Holy Quran states that mothers have to breastfeed their children for two full years, which may be the most important factor that stimulates a positive attitude toward EBF [34]. This cultural and religious relation to BF is also confirmed by some Saudi studies, which reflect a positive attitude toward BF [12]. Although, BF attitudes differ greatly according to age, religion, and some demographic variables [35].

On the contrary, Yasser Abulreesh et al. reported a negative attitude toward EBF among their participants [36]. The variation between the current study and Yasser Abulreesh et al. may be attributed to the different study samples where all their participants were working women, but in the current study, 44.5% were housewives. The housewife was found to have higher EBF intention from binary logistic regression when taking a working woman as a reference in the current study. Moreover, around one-half of the current study participants had a high intention for EBF. Previous Saudi studies also reported high EBF intention [16,29,37]. A higher intention for EBF was reported in Galicia (Spain) [38], Morocco [39], Brazil, Taiwan, Thailand, South Korea, and the United Kingdom [40].

The current study results showed that the most important modifiable predictors of EBF intention among pregnant women were occupational status, previous BF practice, BF knowledge, and attitude. Employed mothers were four times less likely to intend to exclusively breastfeed compared to housewives, likely due to their lack of time. This is particularly relevant in Saudi Arabia, where the maternity leave period is only 45–70 days, making it difficult for employed mothers to exclusively breastfeed for six months without hiring babysitters. Similar findings were reported in Central Ethiopia, where employed mothers were less likely to practice exclusive breastfeeding than non-working mothers [41]. Considering these prior challenges, many researchers have recommended paid maternity leave, proper institutional policies, and BF breaks, as they are the most important interventions in enhancing EBF practice among employed mothers [42,43,44]. On the contrary, Awoke et al. stated that employed women had a higher probability of practicing optimal breastfeeding when compared with housewives. They explained that breastfeeding leaves from work were significantly enhanced in South Ethiopia [45]. Although breastfeeding leave is also applied in Saudi Arabia, housewives still have a higher probability of practicing optimal breastfeeding.

The results of the current study showed that previous BF experience is one of the most impactful predictors of encouraging pregnant women to exclusively breastfeed their newborns. Women with previous experience with EBF or nonexclusive BF have a higher probability of EBF intention when compared with women who never breastfeed. This result is in line with studies by Fernandes and Höfelmann and DiGirolamo et al. They stated that previous BF experiences positively impact EBF intention, initiation, and duration [46,47].

The present study showed that good BF knowledge and positive maternal attitude toward BF are the most important associated factors with a stronger intention to EBF. The findings also showed that pregnant women with adequate knowledge had a stronger intention to EBF. These results highlight the need for BF educational interventions that strongly focus on prenatal education, and the consequent acquisition of adequate knowledge and positive attitudes toward the BF benefits are important to increase the rate of EBF practices. The current study’s findings align with previous research that has shown a correlation between knowledge and attitude toward BF and the intention to exclusively breastfeed [16,47,48,49,50]. They showed a significant relationship between mothers’ overall BF knowledge and attitude and their intention to breastfeed exclusively. They found that mothers with inadequate BF knowledge and restrictive attitude had less intention to EBF than their peers with adequate knowledge, well-informed about the EBF benefits, and positive attitudes. In addition, Plagens-Rotman et al. emphasized the significant role midwives and physicians can play in increasing maternal knowledge regarding EBF. They further highlighted that most of their participants considered breastfeeding as a priority for the infant’s health [51]. The present study determined the positive predictors for high EBF intention. These predictors are very important during antenatal education and counseling regarding BF.

Other modifiable factors, such as antenatal care, plan for the current pregnancy, mode of delivery of the last child, and pregnancy-related complications, are potential predictors for EBF intention. Regular ANC is another factor that increases the likelihood of higher EBF intention. This may be attributed to the proper counseling through prenatal classes for pregnant women about the benefits of EBF. Thus, they are more likely to have higher intentions for EBF than their peers who did not receive ANC and counseling. In this regard, Agho et al. reported that pregnant women who received ANC services were more likely to practice EBF as important key messages are regularly provided during ANC visits [52]. Also, Senghore et al. evaluated the determinant of EBF knowledge and intention during the ante-natal and postnatal periods. They reported that the participants who received counseling during pregnancy on EBF were found to have better odds of intending EBF than those without counseling [53]. Therefore, it is important to include EBF-related information as an essential part of antenatal and postnatal education to promote the EBF practice [54]. ANC breastfeeding counseling should be supported by baby-friendly policies, and mothers should be aware of such policies. Kasem et al. reported that the availability and mother awareness of breastfeeding-friendly policies significantly enhanced women’s perception and initiation of EBF [55].

The results further indicated that planning for the current pregnancy was found to be a significant predictor of high EBF intention. This may be because a woman with an unplanned pregnancy experience psychosocial stress that prevents her from engaging in desired public health behaviors such as adherence to EBF. This result is in agreement with Balogun et al., Hromi-Fiedler et al., and Lau, who stated that unplanned pregnancy decreases the likelihood of having a high intention to EBF and greatly affect BF behavior [56,57,58]. Consequently, maternity nurses and midwives should screen all pregnant mothers for unintended pregnancy to offer them supportive care and counseling during ANC to increase their EBF intention.

The association between the mode of delivery and the IFI score was an important result reported in the current study. Women with a history of vaginal delivery had higher EBF intention when compared with nulliparous women. However, cesarean section was not associated with EBF intention. A recent Saudi study illustrated that the mode of delivery is an important associated factor with the early introduction of supplementary feeding. Since women who had a cesarean delivery were four times more likely to introduce complementary feeding after the first three months of infant life [59], another study in the Saudi context aimed to investigate the predictors of early introduction of supplementary feeding found a cesarean delivery to be influencing factor concerning the initial introduction of supplementary feeding [60]. 

### Strengths and Limitations

All instruments used for data collection in the current study were standardized and validated in previous methodological research, which increased the credibility of the data [16,18,19]. The study had several strengths, including a calculated sample size based on a 95% confidence interval and the use of systemic random sampling to recruit participants, which increased the generalizability of the data. The adjusted Odds ratio was also used to accurately estimate the role of each predictor in EBF intention. However, there were some limitations, such as the reliance on self-reported data, which could be biased by recall or social acceptance. Additionally, the study only collected data from one region in Saudi Arabia, although similar positive predictors have been reported in other studies [12,37,45]. The current study examines the BF intention among pregnant women without follow-up for BF practices during the postpartum period. Therefore, further study is recommended to follow the pregnant women until postpartum to correlate BF intention and EBF practices.

## 5. Conclusions

The current study results concluded the most important modifiable predictors of EBF intention among pregnant women were occupational status, previous BF practice, BF knowledge, and attitude. Other modifiable factors, such as antenatal care, plan for the current pregnancy, mode of delivery of the last child, and pregnancy-related complications, are potential predictors for EBF intention. Data provided from this study can guide healthcare providers’ educational efforts to increase EBF intention among expectant mothers focusing on the identified predictors. Furthermore, community leaders and stakeholders can use data addressed in the current study to help behavior change, communicate culturally appropriate intervention, and access target high-risk groups guided by the identified predictors. 

## Figures and Tables

**Table 1:** Demographic characteristics of the study participants (*n* = 382).

Demographic Characteristics	*n* = (382)	%
**Age** (years)		
<20 years	30	7.8
20–35	294	77.0
≥36	58	15.2
**Mean ± SD**	27.74 ± 6.74	
**Residence**		
Rural	20	5.2
Urban	362	94.8
**Occupation**		
Housewife	170	44.5
Working	212	55.5
**Woman education**		
Illiterate	11	2.9
Read and write	66	17.2
Secondary school	189	49.5
University or postgraduate	116	30.4
**Husband education**		
Illiterate	29	7.6
Read and write	81	21.2
Secondary school	112	29.3
University or postgraduate	160	41.9
**Monthly income**		
Not enough	131	34.3
Enough	215	56.3
Enough and save	36	9.4

**Table 2:** Participants’ obstetric and breastfeeding history of the (*n* = 382).

Obstetric and Breastfeeding History	*n* = (382)	%
**Gestational age for current pregnancy:**		
First trimester (1–<13 weeks)	141	36.9
Second trimester (13–<26)	80	20.9
Third trimester (26 and more)	161	42.2
**ANC**		
Regular	326	85.3
Irregular	56	14.7
**Gravidity**		
Primigravida	131	34.3
Multigravida	251	65.7
**Current pregnancy planning**		
Yes	232	60.7
No	150	39.3
**Pregnancy-related complication**		
Yes	51	13.4
No	331	86.6
**Previous breastfeeding practice (*n* = 251)**		
I never breastfeed at all	18	7.2
Exclusive	67	26.7
Nonexclusive	166	66.1
**Last child delivery mode (*n* = 251)**		
Vaginal delivery	139	55.4
Cesarean section	112	44.6
**Number of living children (mean ± SD)**	2.40 ± 2.12	

ANC: Antenatal Care.

**Table 3:** Participants’ total knowledge, attitude, and intention toward EBF (*n* = 382).

Variables	*n* = (382)	%
**Total knowledge**		
Inadequate (18–54)	184	48.2
Adequate (55–90)	198	51.8
Mean ± SD	66.13 ± 12.15	
**Total attitude**		
Negative (17–51)	92	24.1
Positive (52–85)	290	75.9
Mean ± SD	56.32 ± 8.60	
**Total intention**		
Low (0–8)	167	43.7
High (9–16)	215	56.3
Mean ± SD	11.88 ± 2.96	

**Table 4:** Binary logistic regression analysis of the predictors of the EBF intention.

Predictors	EBF Intention	
	AOR (95% CI)	*p*
**Residence**		
Rural	Ref	
Urban	0.682 (0.110–4.240)	0.681
**Occupation**		
Working	Ref	
Housewife	4.216 (1.906–9.326)	0.000 **
**Education**		0.743
University or postgraduate	Ref	
Secondary school	0.191 (0.021–1.727)	0.141
Read and write	0.169 (0.018–1.581	0.119
Illiterate	0.187 (0.043–1.287)	0.191
**Husband education**		0.199
University or postgraduate	Ref	
Secondary school	0.436 (0.102–1.864)	0.263
Read and write	0.864 (0.202–3.701)	0.844
Illiterate	0.675 (0.312–2.602)	0.768
**Monthly income**		0.695
Enough and save	Ref	
Enough	0.858 (0.382–1.926)	0.710
Not enough	1.430 (0.360–5.690)	0.611
**Gestational age for current pregnancy:**		0.270
First trimester (1–<13 weeks)	Ref	
Second trimester (13–<26)	1.748 (0.661–4.628)	0.261
Third trimester (26 and more)	1.770 (0.847–3.698)	0.129
**ANC**		
Irregular	Ref	
Regular	1.718 (0.631–4.678)	0.028 *
**Number of pregnancies (gravidity)**		
Primigravida	Ref	
Multigravida	1.072 (0.780–1.474)	0.669
**Plan for the current pregnancy**		
No		
Yes	1.885 (0.966–3.676)	0.042 *
**Breastfeeding practice (*n* = 251)**		0.000 **
I never breastfeed at all	Ref	
Exclusive	4.284 (1.896–9.679)	0.000 **
Nonexclusive	2.284 (1.652–7.234)	0.005 *
**Mode of delivery of the last child (*n* = 251)**		0.012 *
Never delivered	Ref	
Vaginal delivery	6.628 (1.862–23.586)	0.003 *
Cesarean section	1.649 (0.735–3.701)	0.225
**Pregnancy-related complications**		
Yes	Ref	
No	3.923 (1.297–11.867)	0.15 *
**Age (years)**	1.007 (0.940–1.078)	0.852
**Total EBF knowledge**	1.193 (1.183–1.421)	0.009 *
**Total EBF attitude**	1.282 (1.205–1.365)	0.000 **
**Gravidity**	1.072 (0.780–1.474)	0.669
**Number of living children**	0.825 (0.599–1.137)	0.239
**−2 Log likelihood (282.339a)**	Cox and Snell R Square (0.468)	Nagelkerke R Square (0.628)

AOR: Adjusted Odd Ratio CI: Confidence Interval * significant at *p* ˂ 0.05 ** significant at *p* ˂ 0.001.

## Data Availability

The article describes all data related to this study.

## Abbreviations

ANC	Antenatal Care
BF	Breastfeeding
EBF	Exclusive Breastfeeding
GFBKS	Gender-Friendly Breastfeeding Knowledge Scale
IFI	Infant Feeding Intention
IIFAS	Infant Feeding Attitude Scale
MCH	Maternal and Children Hospital
WHO	World Health Organization
